# Supplementary material for: A Pentavalent HIV-1 Subtype C Vaccine Containing Computationally Selected gp120 Strains Improves the Breadth of V1V2 Region Responses
Source: Vaccines (Basel). 2025 Jan 28;13(2):133. doi: 10.3390/vaccines13020133 (PMC11860947; doi:10.3390/vaccines13020133)
Supplement: Supplementary file 1 [file vaccines-13-00133-s001.zip › vaccines-3386016-supplementary.pdf]

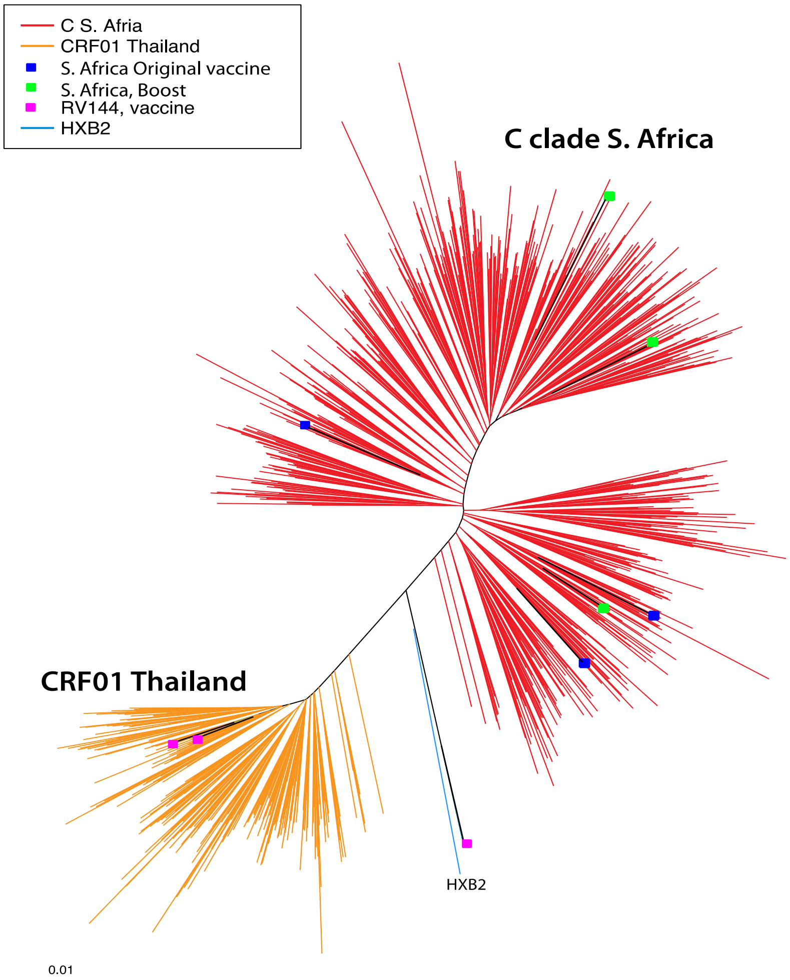

LANL database circa 2013:
245 CRF01 Thai, versus 623 S. African C clade.
Average AA distance (excluding gaps, conservative, Thai vaccine
to circulating Thai CRF01's: 16-17%, South African Cs to C vaccines, 21-23%
(ALSO SEE: Rademeyer, et al., PloS Pathog. 12(7):e1005742, 2016

Figure S1. Diversity of subtype C and AE HIV-1 Env. The average AA distances (excluding gaps) between the 6 subtype C vaccine strains and the SA acute sequences are 22.1, 22.1, 22.8, 22.0, 20.6, and 20.6% for 96ZM651, 1086C, TV1, CAP260, CAP174, and Ko224, respectively. The average AA distances (excluding gaps) between the 2 RV144 subtype AE vaccine strains and the acute AE sequences are 17.5 and 16.2% respectively for A244 and 92TH023. Average distances between the subtype C vaccine strains and acute C sequences are given in Fig 1.

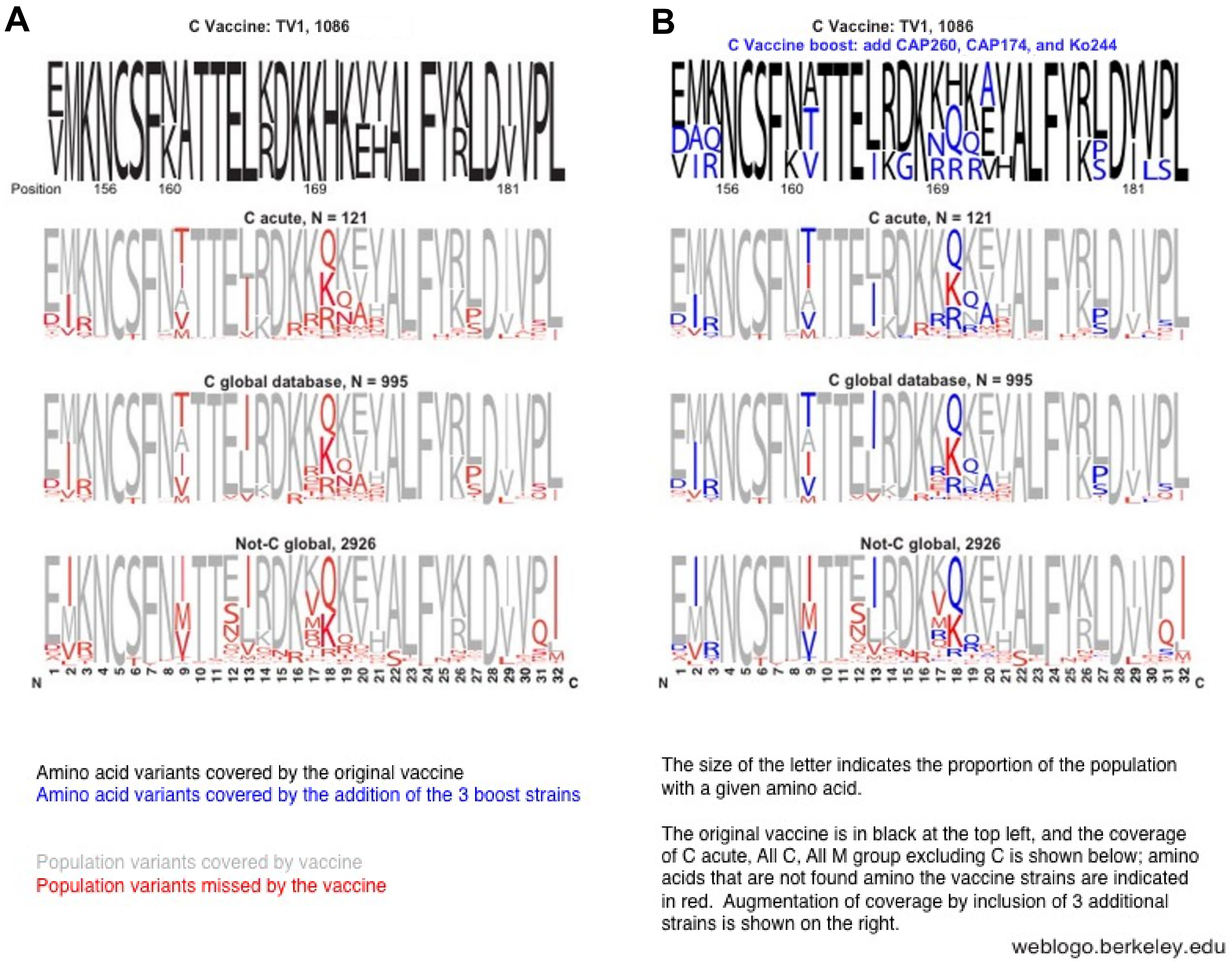

Figure S2. Coverage of V1V2 epitope region variations by the 2 original subtype C P5 boost strains (A) and the 2 P5 vaccine strains plus 3 newly selected boost strains (B). The relative sizes of each amino acid reflects the proportion of the variant in the virus population as indicated (C acute, C global, and Not-C global from top to bottom). Amino acids in red are the variations not covered by the respective vaccine strain combinations. Amino acids in blue are the ones not covered by the 2 original P5 boost strains but are covered by the 3 newly selected boost strains.

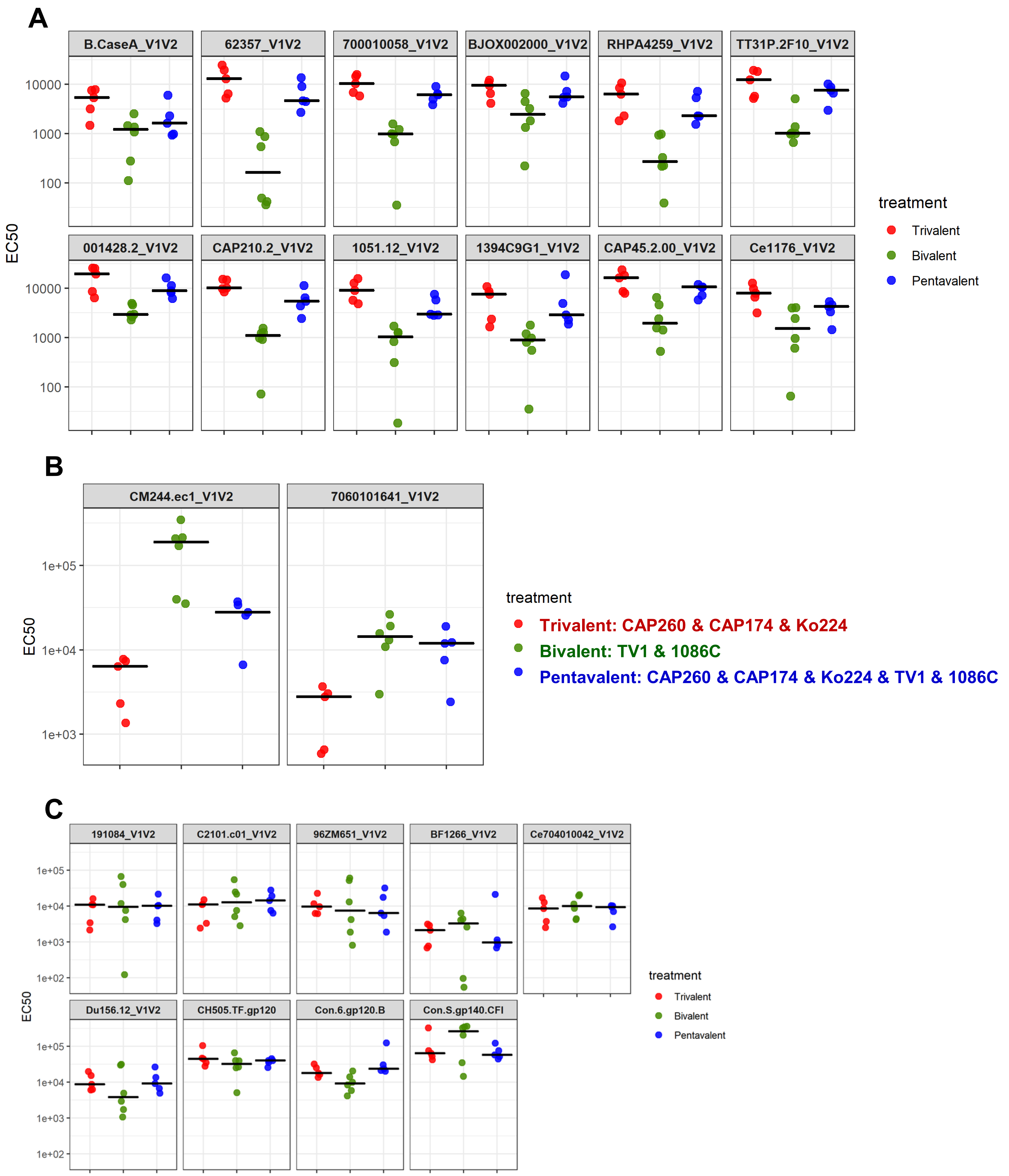

Figure S3. Antibody titers (EC_{50}) measured in BAMA for V1V2 gp70 scaffolds that showed higher (A), lower (B) or comparable magnitudes of binding by the trivalent group compared to the bivalent group. Spots represent individual animals and are color-coded by group. The subtype information for each scaffold are labeled on each plot.

Trivalent: CAP260 & CAP174 & Ko224
Bivalent: TV1 & 1086C
Pentavalent: CAP260 & CAP174 & Ko224 & TV1 & 1086C

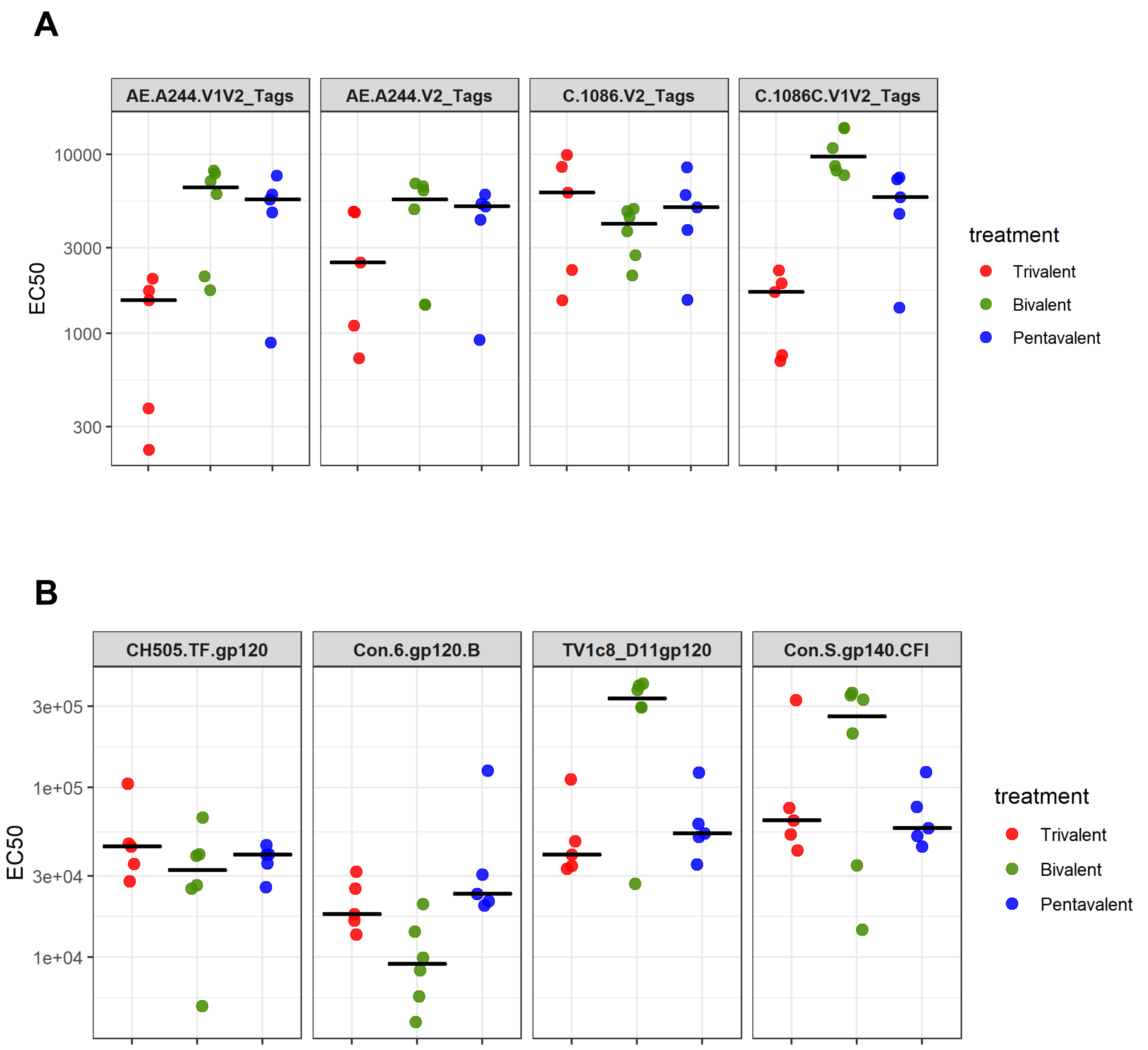

Figure S4. Antibody titers (EC_{50}) measured in BAMA for V2 and V1V2 Tags constructs (**A**) and gp120 and gp140 Env Proteins (**B**) by vaccine groups. Spots represent individual animals and are color-coded by group. The subtype information for each scaffold, and FDR_p value, if <0.05, are labeled on each plot.

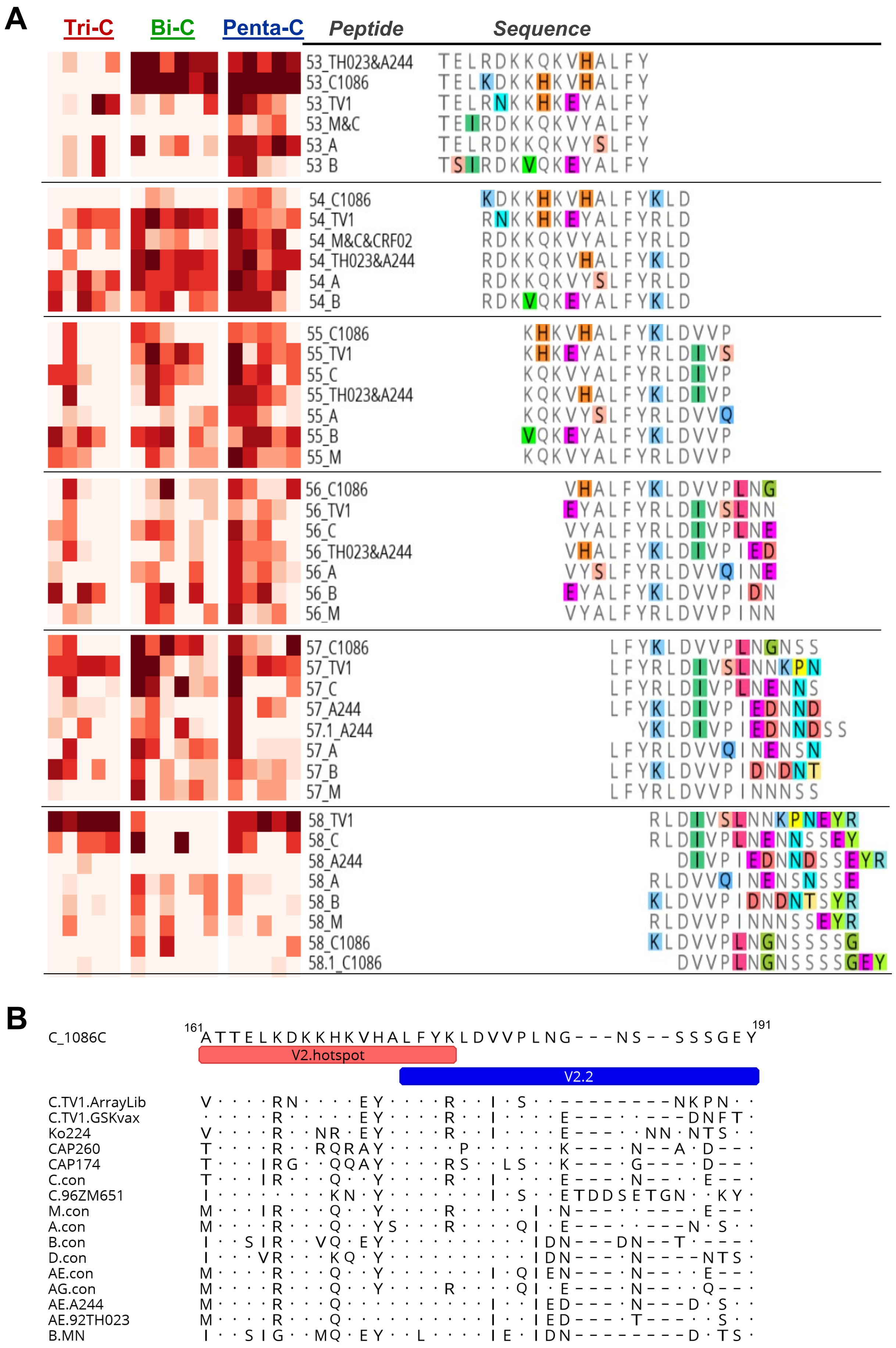

Figure S5. Magnitude of binding (Log2 Fold post-/pre-immunization) to overlapping peptides within V2 region by individual animals (**A**) and alignment of vaccine strain and array library sequences for the V2 linear epitope region (**B**). Each column represents responses for an individual animal to different peptides (indicated as rows). Peptides are filtered for positivity rate >33% in any group. Darker red indicates higher magnitude.

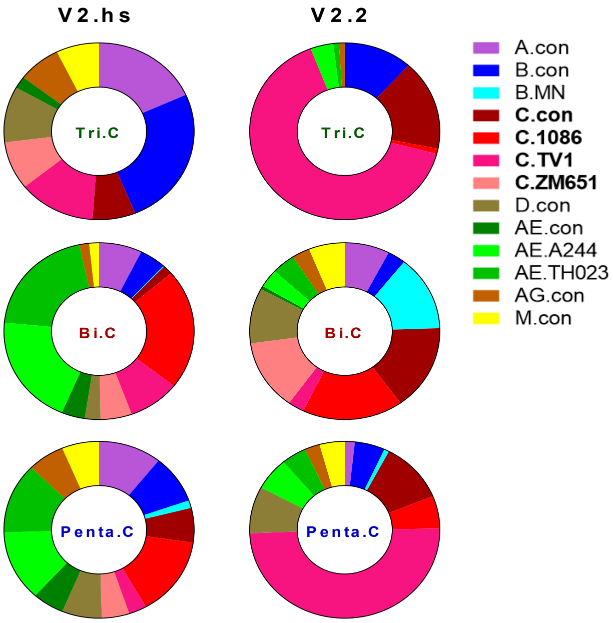

Figure S6. Contributions of Env strains in the library to binding responses targeting V2.hotspot and V2.2 linear epitopes. Each donut chart depicts the overall binding to V2.hotspot (top) or V2.2 (bottom) epitope by one vaccine group (as indicated in center of donuts), with each slice represents relative binding intensity to a specified strain. Binding intensity values plotted are median values per group.

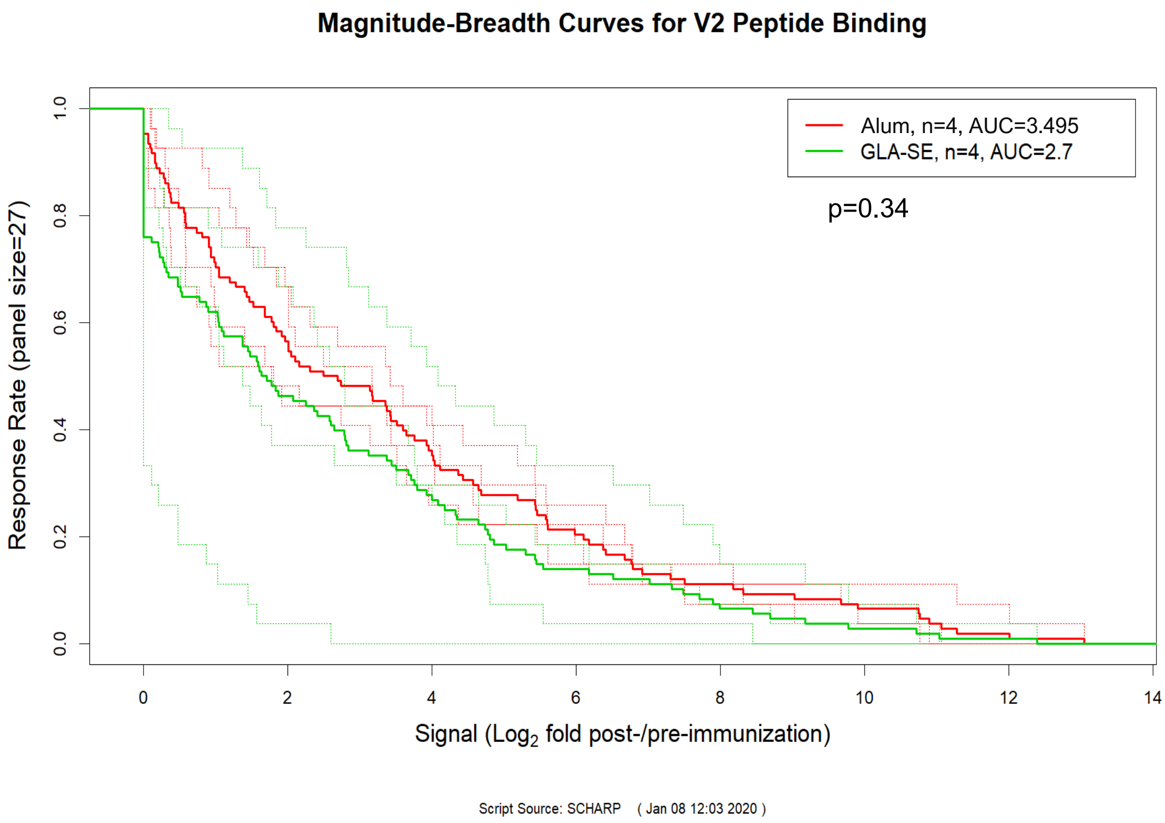

Figure S7. NHP148 magnitude-breadth of binding to V2 linear peptides, by array mapping. Magnitude-Breadth curves for binding to V2 linear peptides for the 2 macaque pentavalent E/C vaccine groups. Breadth is summarized over the overlapping peptides in V2 epitope region with positivity rate >33% for at least one vaccine group (27 peptides total). Each curve is plotted as the proportion the of 27 V2 peptides that the animal responded with a magnitude (Log_2 fold post-/pre-immunization) within the interval indicated on the x-axis. Thin dotted lines represent individual animals, and thick solid lines represent the group median values.

Table S1A. NHP study group median binding antibody titer (EC50) for V1V2 and Env antigen in BAMA and statistical test results for comparison of binding magnitude among groups.

		Group Median EC50		P value*, Wilcoxon Rank Sum test
Category	Analyte	GLA-SE	Alum	GLA-SE vs Alum
Heterologous V1V2 Scaffold	gp70-001428.2.42 V1V2	64	295	**0.03**
	gp70-1051.12.C22 V1V2	210	923	0.20
	gp70-1394C9G1 V1V2	174	1,123	0.11
	gp70-191084_B7 V1V2	192	2,012	0.06
	gp70-62357.14 V1V2	23	150	0.11
	gp70-700010058 V1V2	135	268	0.34
	gp70-7060101641 V1V2	77	1,115	**0.03**
	gp70-96ZM651.02 V1v2	521	1,007	**0.03**
	gp70-BF1266_431a_V1V2	288	1,376	0.11
	gp70-BJOX002000.03.2 V1V2	63	530	0.20
	gp70-C2101.c01_V1V2	293	961	0.11
	gp70-CAP210.2.00.E8 V1V2	197	501	0.49
	gp70-CAP45.2.00.G3 V1V2	325	707	0.20
	gp70-CM244.ec1 V1V2	1,556	7,866	**0.03**
	gp70-Ce1176 V1V2	83	620	0.20
	gp70-Ce704010042_2ES V1V2	146	1,095	0.11
	gp70-Du156.12 V1V2	43	491	0.06
	gp70-RHPA4259.7 V1V2	49	333	0.34
	gp70-TT31P.2F10.2792 V1V2	113	649	0.20
	gp70_B.CaseA_V1_V2	395	1,110	0.34
Vaccine-matched V1V2	gp70-Ce1086_B2 V1V2	1,147	5,629	**0.03**
	gp70-TV1.21 V1V2	531	1,271	0.20
	gp70-TV1.GSKvacV1V2/293F	653	1,183	0.49
V1V2 and V2 Tag	AE.A244 V1V2 Tags/293F	568	1,929	**0.03**
	AE.A244 V2 tags/293F	65	801	0.06
	C.1086 V2 tags/293F	56	170	**0.03**
	C.1086C_V1_V2 Tags	112	1,519	0.06
Envs	CH505 TF gp120	926	1,227	0.34
	Con 6 gp120/B	529	487	0.69
	Con S gp140 CFI	2,323	2,353	0.69
	TV1c8_D11gp120.avi/293F	1,759	2,058	0.49

Table S1B. Statistical test results for comparison magnitude of antibody blocking activity (AUC over the time course) in the two groups of the NHP study.

		P value*, Wilcoxon Rank Sum test
Competiting mAb	HIV Env	GLA-SE vs Alum
CH58	AE.A244_D11gp120/293F/Mon	**0.03**
A32	AE.A244_D11gp120/293F/Mon	0.89

*Raw p values are not adjusted for multiple comparisons. p values <0.05 are bolded.
